# Performance of obturation techniques in anatomical irregularities located at different thirds of the root canal system

**DOI:** 10.1590/1678-7757-2023-0440

**Published:** 2024-05-20

**Authors:** Silverio Vazquez-Alcaraz, Lucia Gancedo-Caravia, Ana Arias, Jaime Bascones

**Affiliations:** 1 Universidad Complutense de Madrid Facultad de Odontología Departamento de Odontología Conservadora y Prótesis España Universidad Complutense de Madrid, Facultad de Odontología, Departamento de Odontología Conservadora y Prótesis, España

**Keywords:** Anatomical irregularities, Gutta-percha filled area, Micro-CT-based tooth replica, Obturation techniques, Root canal anatomy

## Abstract

This study aimed to compare the quality of root canal obturation (ratio of area occupied by gutta-percha (G), sealer (S), and presence of voids (V)) in different anatomical irregularities (intercanal communications, lateral irregularities, and accessory canals) located at different thirds of the root canal system of mandibular molar replicas. Sixty-seven 3D printed replicas of an accessed mandibular molar were prepared using ProGlider and ProTaper Gold rotatory systems. Three specimens were randomly selected to be used as controls and did not receive further treatment. The rest were randomly distributed in 4 experimental groups to be obturated using either cold lateral compaction (LC), continuous wave of condensation (CW), and core-carrier obturation (ThermafilPlus (TH) or GuttaCore (GC)) (n=16 per group). AHPlus^®^ sealer was used in all groups. The three controls and a specimen from each experimental group were scanned using micro-computed tomography. The rest of the replicas were sectioned at the sites of anatomical irregularities and examined at 30× magnification. The G, S, and V ratios were calculated dividing the area occupied with each element by the total root canal area and then compared among groups using the Kruskal-Wallis test. Voids were present in all obturation techniques with ratios from 0.01 to 0.15. CW obtained a significantly higher G ratio in the irregularity located in the coronal third (0.882) than LC (0.681), TH (0.773), and GC (0.801) (*p*<0.05). TH and GC achieved significantly higher G ratios in those located in the apical third (*p*<0.05). The worst quality of obturation was observed in the loop accessory canal with all obturation techniques. Whitin the limitations of this study, it can be concluded that CW and core-carrier obturation are respectively the most effective techniques for obturating anatomical irregularities located in the coronal and the apical third.

## Introduction

Irregular internal anatomy and intricacies of the root canal system are a challenging obstacle when it comes to debridement and obturation in endodontic procedures.^1,2,3^ After cleaning and shaping, a three-dimensional sealing of the complete root canal system is essential to entomb remaining bacteria, if existent.^3^ Several techniques have been suggested to fill the root canal system with an adequate ratio of gutta-percha and sealer.^4,5^ However, the quality of obturation might decrease in the presence of anatomical irregularities. Cold lateral compaction (LC) does not always ensure that gutta-percha penetrates in narrow and irregular spaces that may be filled solely with the sealer, or even remain unfilled.^6^ Thermoplastic gutta-percha techniques such as continuous wave of condensation (CW) and core carrier techniques have been recommended for enhancing the quality of obturation of internal irregularities.^7-9^

Extracted human teeth have been widely used to evaluate the quality of root canal obturation using different materials and techniques.^4,5,10^ However, the lack of anatomical standardization limits their use when analyzing internal irregularities. To overcome this drawback, three-dimensional (3D) printed replicas based on microcomputed tomography (micro-CT) scans have been recommended as an alternative for standardization.^11^ To date, very few studies have compared filling techniques in replicas with complex internal anatomy. Few studies that used replicas of molars for this purpose analyzed very specific anatomical singularities, such as band-shaped isthmuses located in the apical third of roots^12^ and C-shaped canals.^9^ No previous studies have determined the filling capacity of the most common techniques in replicas of molars with anatomical irregularities at different levels of the root canal system.

Therefore, this study aims to compare the quality of root canal obturation (ratio of areas filled with G or S and presence of V), by analyzing different techniques in anatomical irregularities at the different thirds of the root canal system, using replicas of mandibular molars. The null hypothesis tested was that there is no significant difference in the G, S, and V ratios produced by different obturation techniques in anatomical irregularities at different locations.

## Methodology

### Sample selection

**Figure 1:**
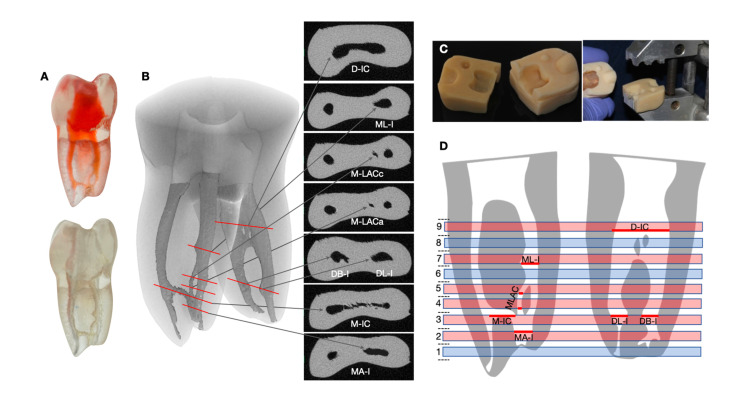
TrueToothTM replica before (top) and after (bottom) instrumentation. (B) Three-dimensional reconstruction of a non-obturated micro-CT-scanned replica and cross-sectional images of the irregularities (red lines indicate location). (C) Images of the fixing device built to assemble the replica. (D) Schematic representation of the longitudinal view of the mesial (left) and distal (right) roots, illustrating the sections location. Red lines on selected sections highlight the evaluated areas of interest: D-IC (distal intercanal communication), ML-I (mesial lateral irregularity), M-LACc / M-LACa (mesial accessory loop canal (coronal and apical sections)), DB-I (disto-buccal lateral irregularity), DL-I (disto-lingual lateral irregularity), M-IC (mesial intercanal communication), MA-I (mesial apical irregularity)

A commercially available 3D printed, transparent, radio-opaque tooth replica (TrueTooth™ 30-01, Dental Engineering Laboratories, Santa Barbara, CA, USA) was used in this study. It was obtained by micro-CT scanning a mandibular first molar (Figure 1A) from a human that was previously accessed.

For sample size calculation, a power of 0.90 and an α-type error of 0.05, were set to detect an effect size of 0.51, based on a pilot study data. A total sample size of 60 was estimated, with 15 specimens per group. Sixty-seven specimens were used (three served as controls and 64 were randomly allocated to four experimental groups, as described later.)

The tooth presented intercanal communications, accessory canals, and lateral irregularities based on the coding system suggested by Ahmed, et al.^13^ (2021) (Figure 1B). In detail, the mesial root had two main canals (mesio-buccal, MB; and mesio-lingual, ML) connected by an intercanal communication in the apical third (M-IC). The ML root canal presented a band-shaped lateral irregularity in the apical portion (mesial apical irregularity, M-AI), a loop acessory canal (mesial loop accessory canal M-LAC), and an oval-shaped lateral irregularity (mesial lateral irregularity, M-LI) in the middle third. The distal root presented a 1-2 configuration with a large canal in the coronal third that bifurcated into a disto-buccal (DB) and a disto-lingual (DL) canal at mid-root with oval-shaped lateral irregularities in the apical third of both canals (disto-buccal irregularity, DB-I; and disto-lingual irregularity, DL-I) and an intercanal communication (distal intercanal communication, D-IC) in the coronal third. Figure 1 shows the precise location of anatomical irregularities. Apical diameters were 0.4 and 0.5 mm for mesial and distal root canals, respectively.^14^

### Root canal preparation

All root canal treatments were performed under a stereoscopic microscope at 10× magnification (Leica 541, Leica Microsystem, Wetzlar, Germany) by a single operator with >20 years of experience in endodontics (J.B.). Root canal length was determined by inserting a size #10 K-file (Dentsply Sirona, Ballaigues, Switzerland) into each root canal until the tip was visible at the apical foramen, and then the working length (WL) was established by subtracting 1 mm from the recorded length. Root canal preparation was performed using the ProGlider^®^ and ProTaper Gold^®^ rotatory system (Dentsply Sirona) following the manufacturer’s instructions for use, up to an F4 file in the mesial canals and F5 in the distal canals. After each instrument, the root canal was irrigated with 2 ml of 5.25% sodium hypochlorite (DentaFlux, Madrid, Spain), using a syringe with a 27gauge side-vented needle (Monoject Endodontic Irrigation Syringe; Covidien, Mansfield, MA, USA). Apical patency was maintained by inserting a 10 K-file 1 mm beyond the apical foramen. Stainless-steel hand K-files #40 and #50 were used in the mesial and distal canals, respectively, to gauge the apical preparation. After final irrigation with 3 ml distilled water in each canal, the root canals were dried with paper points.

### Root canal obturation – experimental groups

Three specimens were randomly selected to be used as controls after shaping and did not receive further treatment. The remaining 64 specimens were randomly allocated into four groups (n=16) based on obturation technique to be used: cold lateral compaction (LC), continuous wave of condensation (CW) and core carrier obturation with Thermafil Plus^®^ (TH) and GuttaCore^®^ (GC) (Dentsply Sirona). In all groups, 0.05 ml of AHPlus^®^ sealer (Dentsply Sirona) was applied with a #40/.02 paper point (Dentsply Sirona) to coat the root canal walls, and the root canals were filled following the same sequential order: ML, MB, DL and DB.

### Group LC: Cold lateral compaction

Standardized #40.02 and #50.02 master gutta-percha cones (Dentsply Sirona) were used respectively for the mesial and distal root canals, according to the apical preparation. One master cone was inserted into each root canal to WL. XF accessory cones (Dentsply Sirona) cones (Dentsply Sirona) were then laterally compacted until a size B nickel-titanium Finger Spreader (Dentsply Sirona) could no longer penetrate the coronal third of the root canal. Approximately 25 accessory gutta-percha cones were used for each replica (10 in the mesial canal and 15 in the distal canal). Excess material was removed from the orifice.

### Group CW: Continuous wave of condensation

F4 and F5 Protaper Gold gutta-percha cones (Dentsply Sirona) were used for the mesial and distal root canals, respectively, according to the apical preparation. The gutta-percha was fitted to the WL and down-packed using a System B heat carrier and a .06 F plugger (Elements Obturation System, SybronEndo, Orange, CA, USA) that was inserted 4 mm short from the WL. The heat carrier was set at a temperature of 175 °C, as recommended by TrueToothTM manufacturer.^14^ A Buchanan Hand Plugger Size 1 (Sybron Endo) was then used to compact the gutta-percha in the apical third of the root. It was then backfilled with 3-4 mm increments of warm injected gutta-percha with the Extruder handpiece (Elements Obturation System, SybronEndo), and the gutta-percha was compacted with the same plugger.

### Groups TH and GC: Core-carrier obturation with Thermafil Plus and GuttaCore

Both core carrier techniques (Thermafil Plus and GuttaCore, Dentsply Sirona) were performed according to manufacturer’s instructions. Sizes 35 and 45 were selected based on size confirmation using verifiers (Dentsply) for the mesial and distal root canals, respectively. Thermafil obturators were heated in a Thermaprep Oven^®^ (Dentsply Sirona) whereas GuttaCore obturators were heated in a GuttaCore Oven^®^ (Dentsply Sirona). The softened obturators were inserted at 3 mm/s into the canal to WL and maintained and maintained under light apical pressure for 8 s.

All samples were kept at room temperature for two weeks to allow complete setting of the sealer.

### Root sectioning

A fixing device was built to ensure uniform orientation of tooth replicas during sectioning. For this purpose, the crown was immersed in a block of acrylic resin (Trim, Bosworth Company, East Providence, RI, USA) and assembled in the clamps of a sewing machine (Exakt Cutting Unit 400C, Exakt Advanced Technologies, Norderstedt, Germany) (Figure 1C). Roots were sectioned under copious irrigation with water at 5º C. Nine consecutive 0.7-mm-thick sections were obtained from each tooth, with the first cutting site located 1 mm coronal to the MB apical terminus. Sections 2, 3, 4, 5, 7, and 9 allowed the evaluation of anatomical internal irregularities, as shown in Figure 1D. The apical and coronal portions of the loop accessory canal were evaluated in two different sections (M-LACa and M-LACc, respectively).

### Evaluation and analysis

Images of the slices were obtained at 30× magnification using a digital camera (Leica EC3, Heerbrugg, Switzerland) connected to a stereomicroscope (Nikon SMZ800 Stereo Zoom Microscope, New York, NY, USA). A single calibrated operator, blinded to the experimental groups, performed all measurements. The cross-sectional area of the root canal and the area filled with G, S, and presence of V were measured for all irregularities using ImageJ 1.51h program (U. S. National Institutes of Health, Bethesda, MD, USA). The resulting values were formulated as ratios, dividing the area occupied with each element (G, S, and V) by the total root canal area. The data distribution of several groups was not compliant with normality; therefore, the nonparametric Kruskal-Wallis test was used to compare the ratio of areas filled with G, S, and presence of V for each internal irregularity among groups. SPSS Statistics 25.0 program (IBM, New York, NY, USA) was used for the statistical analysis. The level of significance was set at p<0.05.

The three unfilled replicas and one additional representative specimen from each experimental group were scanned using X-ray micro-CT (CT-SCAN-XT H-160, NIKON METROLOGY NV, Leuven, Belgium) with voxel size of 16.6 μm, 134 kV, 237 μA, 1 mm aluminum and 0.25 mm copper filters. The sample stage rotated 360° and collected 3015 images and two frames per scan. Dragonfly 3D imaging program (Object Research Systems, Montreal, QC, Canada) was used to import the TIFF images and to create datasets for internal anatomy segmentation and modelling, as described by Holmes, et al.^15^ (2021) (Figure 1B). A threshold value was defined according to the range of gray shades corresponding to empty space (controls and V) as well as obturation materials (G and S). A multilayer mask was created containing the complete root canal system, from the cementoenamel junction to the apical foramen. Subsequently, the total volume of the mask was calculated. 2D cross-sectional images were obtained from 0.02 mm micro-CT slides. The exact locations of the different slides were established according to the previously described sectioning sites. The area of each element (G, S, and V) detected in the 2D images was measured, and the ratios of the areas filled with G, S, and presence of V were then calculated for reference.

## Results

Mean total volume and standard deviation (SD) of the root canal was 38.94 (0.25) mm^3^ for the control replicas and 38.7 (0.46) mm^3^ for the representative specimens of the experimental groups. The median and interquartile range (IQR) of G, S, and V ratios for the four obturation techniques and all irregularities are presented in Table 1, as well as the reference ratios obtained from the micro-CT analysis. Statistically significant differences were observed among the groups in the ratios of G and S in all the irregularities, except for M-LACa. Groups showing higher ratios of G varied depending on the location of the anatomical irregularity.

**Table 1:** Median ratios and (interquartile range (IQR)) of areas represented by gutta-percha (G), sealer (S) and voids (V) obtained with sectioning (and reference micro-CT ratio) for each obturation technique (LC (lateral compaction), CW (continuous wave of condensation), TH (Thermafil Plus), GC (GuttaCore)) at the different anatomical irregularities

ANATOMICAL IRREGULARITY																									
MESIAL ROOT													DISTAL ROOT												
APICAL THIRD													MIDDLE THIRD						APICAL THIRD			CORONAL THIRD			
	Distance to respective apex (mm)	1			2.5			3.5			4 (to MB) 1.5 (to ML)			6			3			3			7.5 (to DB) 8 (to DL)		
		M - AI			M - LACa			M - LACc			M - IC			M - LI			DB - I			DL - I			D - IC		
	Group (obturation technique)	Median value (IQR) | reference microCT ratio																							
**Ratio of area filled with G**	LC	0.25^b^	(0.05)	0.24	0.00	(0.00)	0.00	0.00^b^	(0.00)	0.00	0.33^c^	(0.09)	0.33	0.51^b^	(0.14)	0.55	0.63^b^	(0.17)	0.55	0.56^b^	(0.11)	0.52	0.68^c^	(0.08)	0.66
	CW	0.18^b^	(0.04)	0.18	0.00	(0.18)	0.00	0.49^a^	(0.50)	0.44	0.47^b,c^	(0.13)	0.55	0.85^a^	(0.11)	0.85	0.54^b^	(0.18)	0.51	0.60^b^	(0.17)	0.62	0.88^b^	(0.06)	0.88
	TH	0.61^a^	(0.27)	0.59	0.00	(0.51)	0.63	0.57^a^	(0.25)	0.50	0.65^a,b^	(0.28)	0.59	0.88^a^	(0.05)	0.87	0.71^a^	(0.14)	0.71	0.74^a^	(0.09)	0.73	0.77^a^	(0.07)	0.75
	GC	0.47^a^	(0.14)	0.46	0.00	(0.48)	0.75	0.49^a^	(0.34)	0.78	0.69^a^	(0.09)	0.67	0.76^a^	(0.20)	0.77	0.73^a^	(0.10)	0.68	0.67^a^	(0.16)	0.7	0.80^a^	(0.10)	0.78
**Ratio of area filled with S**	LC	0.69^a^	(0.09)	0.71	0.85	(0.29)	0.96	0.94^a^	(0.22)	0.99	0.59a	(0.13)	0.32	0.41^a^	(0.11)	0.42	0.31^a,b^	(0.15)	0.45	0.32^a,b^	(0.16)	0.45	0.29^a^	(0.05)	0.33
	CW	0.7^a^	(0.22)	0.62	0.93	(0.55)	0.85	0.37^b^	(0.41)	0.22	0.45^b^	(0.11)	0.34	0.12^b^	(0.08)	0.15	0.36^a^	(0.12)	0.43	0.36^a^	(0.17)	0.38	0.09^c^	(0.09)	0.11
	TH	0.32^b^	(0.20)	0.36	0.63	(0.55)	0.25	0.30^b^	(0.25)	0.20	0.32^c^	(0.23)	0.41	0.08^b^	(0.08)	0.13	0.25^b^	(0.15)	0.26	0.21^c^	(0.11)	0.23	0.19^b^	(0.09)	0.24
	GC	0.46^b^	(0.20)	0.44	0.61	(0.63)	0.21	0.43^b^	(0.33)	0.11	0.29^c^	(0.10)	0.3	0.18^b^	(0.15)	0.21	0.24^b^	(0.17)	0.31	0.24^b,c^	(0.18)	0.29	0.18^b,c^	(0.14)	0.21
**Ratio of area with V**	LC	0.07	(0.10)	0.05	0.15	(0.29)	0.04	0.06	(0.19)	0.01	0.09^a^	(0.09)	0.35	0.08	(0.13)	0.03	0.04	(0.14)	0.01	0.10	(0.14)	0.03	0.03	(0.03)	0.01
	CW	0.11	(0.16)	0.20	0.07	(0.16)	0.15	0.08	(0.14)	0.33	0.04^a,b^	(0.13)	0.11	0.03	(0.05)	0.00	0.07	(0.10)	0.06	0.03	(0.07)	0.00	0.01	(0.04)	0.01
	TH	0.02	(0.09)	0.05	0.09	(0.30)	0.13	0.13	(0.18)	0.3	0.04^a,b^	(0.05)	0.01	0.03	(0.07)	0.00	0.06	(0.08)	0.03	0.03	(0.06)	0.04	0.02	(0.04)	0.00
	GC	0.04	(0.10)	0.10	0.12	(0.19)	0.04	0.11	(0.12)	0.11	0.04^b^	(0.02)	0.03	0.05	(0.11)	0.02	0.02	(0.05)	0.02	0.04	(0.05)	0.01	0.03	(0.04)	0.01

Statistically significant differences among groups in G, S and V ratios (*p*<0.05) are indicated with a,b,c superscripts(a indicates higher ratio than b, and b than c).

In the irregularities located at the apical third (M-AI, M-IC, DB-I, and DL-I), the ratio of G was significantly higher (*p*<0.05) by use of core-carrier obturation techniques (both TH and GC.) The CW group had the lowest G ratio at M-AI (*p*<0.05), showing no significant difference from the LC group. The ratio of sealer was also significantly lower in TH and GC, and no differences were detected in the ratio of voids, except in M-IC, where LC showed a higher ratio of voids. In the middle third of the root (M-LI) the G ratio was significantly lower in LC group than in the other groups (*p*<0.05), with no significant differences among the three thermoplastic techniques. At this location, LC showed a significantly higher V ratio than CW. In the coronal third (D-IC), the CW group showed the highest ratio of G in comparison with the rest of the groups (*p*<0.05), followed by the core-carrier obturation techniques, and no differences were detected in the ratio of voids.

**Figure 2:**
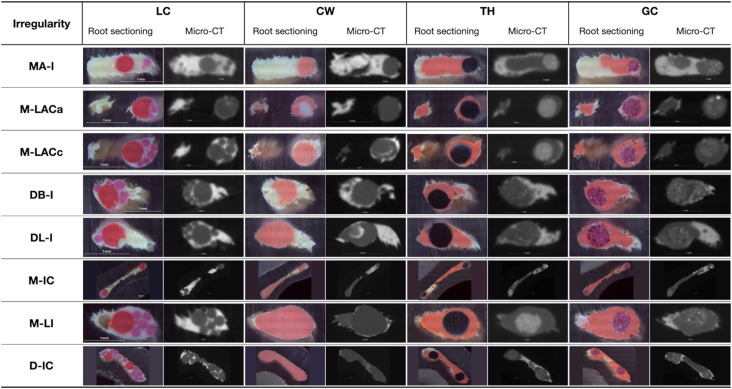
Representative cross-sections of the replicas obtained by sectioning and micro-CT scanning anatomical irregularities, for all obturation techniques: LC (lateral compaction), CW (continuous wave obturation technique), TH (Thermafil Plus), GC (GuttaCore)

The M-LAC irregularity showed low ratios of G-filled area at the apical site (M-LACa), with no significant differences among the four techniques. In M-LACc, the ratio of G was significantly higher for the core carrier and CW obturation techniques (*p*<0.05). Figure 2 shows representative cross sections of the replicas from all anatomical irregularities and groups.

## Discussion

This study was designed to quantify the ratio of G and S, as well as the presence of V in multiple technique obturations of root canals with anatomical irregularities. The null hypothesis was rejected. Significant differences among obturation techniques were found in G and S ratios in all irregularities, except for M-LACa. Differences in V ratios were also detected in M-IC and ML-I. The complex anatomy of many root canal systems challenges clinicians, and inadequate obturation of root canal anomalies has been reported in literature.^1,3,16^ Although gutta-percha has been the main filling material, it does not provide a proper seal,^17^ and a sealer is required to cover the dentin wall, with the smallest thickness possible. According to Gulsahi, et al.^18^ (2007), both the root canal preparation and the obturation technique influence on sealer distribution inside the root canal. Libonati, et al.^10^ (2018) found that excessive sealer can accumulate in narrow anatomical spaces, preventing thermo-plasticized gutta-percha from penetrating. The authors concluded that, although the sealer is essential to any obturation technique, it can also slow down the gutta-percha progression during heating.^10^

This study was performed using 3D-printed tooth replicas obtained from micro-CT scans of a natural tooth to allow sample standardization.^9,12,15^ Although replica usage might be a limitation to this study, the standardization of anatomical irregularities in natural teeth is difficult to achieve. The mandibular molar replica used in this study was selected to ensure a reproducible method for performance comparison of different obturation techniques in different anatomical irregularities located in the apical, middle, and coronal thirds of root canals. Previously published studies have also used reproductions of mandibular molars to evaluate root canal obturation techniques,^9,12^ although they analyzed more limited anatomical irregularities in terms of location and variety than those selected for this experiment.

Another limitation to this study is that the root canal system of the replica was not previously shaped, and each replica was prepared before obturation. This aspect might affect canal volume homogeneity of the different groups before obturation.^19^ Commercially available models are often preferred when obtaining internal anatomies with fine details,^20^ however commercially available replicas with root canal preparations do not present such variety of anatomical irregularities. Also, some strategies were used to try to homogenize the specimens canal volume in the different groups. Firstly, all samples were prepared with the same shaping protocol using ProGlider and ProTaper Gold rotatory systems. In addition, three replicas were micro-CT scanned to be used as controls after shaping. The total volume of the root canal space in the control samples and in the representative specimens of the experimental groups was then calculated showing minor differences among the replicas (from 38.10 to 39.19 mm^3^). This evaluation showed fair consistency in the volume of prepared root canals. Moreover, the replicas were also randomly distributed in 4 experimental groups after being shaped.

In fact, all root canals were originally large, therefore the preparations final diameters might exceed typical recommendations for mesial and distal root canals in clinical situation. This might be another limitation, but on the other hand replicas used in this study were selected due to its internal anatomy complexity. Indeed, root canal obturation in the main root canals was not evaluated. Used instruments are likely to have low impact on the walls of the main root canals.^12^ In addition, the instruments did not reach the anatomical irregularities and therefore, should not have altered their internal anatomy. Still, some shavings could have entered the irregularities when shaping the main root canals of acrylic teeth and may have affected the results by preventing the filling materials to access the irregularities. In accordance with previous studies,^4,5,12,15,16^ this study did not include irrigant solutions activation before obturation in the protocol to avoid possible alterations on the resin walls surface. Activation of irrigant solutions is expected to enhance cleaning and improve obturation techniques performance in teeth;^21^ however, the activation behaves differently in dentin compared to resin replicas.^22^ Studies analyzing the extrusion of irrigants or debris during endodontic cleaning and shaping procedures simulate close-ended systems,^22^ however, it is not a common practice in obturation studies to use models that simulate the effect of periodontal ligament.^19^ This study only analyzed obturation quality in internal anatomical irregularities, and not in the main canal. It also did not simulate a closed-ended system according to the clinical situation; therefore, the results should be interpreted with caution.

Lastly, this study major limitation is that root sectioning is a destructive method that allows evaluation of a limited number of slices per root.^19^ Hence, it is a limited method for analyzing the quality of obturation along the entire root canal. Also, its accuracy has been questioned due to the possible damage and partial loss of the specimens during sectioning.^19^ This study aimed to compare the performance of different obturation techniques in anatomical irregularities exclusively. The locations of the root sectioning sites were selected to enable evaluation of the areas of interest using an affordable method. In addition, the fixing device designed for this study positioned the samples accurately during sectioning to preserve the integrity of critical areas of interest, considering the small size of anatomical irregularities. Special care was taken to prevent the melting of gutta-percha and other possible distortions due to the sawing process with an abundant supply of cold water. A micro-CT of a sample from each experimental group was used as reference, although the reliability of micro-CT analyses for obturation materials with similar densities has also been discussed as limited.^15,19^Figure 2 shows examples of root slices and their corresponding micro-CT virtual sections. Jung, Lommel and Klimek^23^ (2005) also reported a fair correlation between the results obtained from root sectioning and from micro-CT techniques.

Previous studies have reported higher ratios of areas filled with G in oval canals compared to this study^4,5,16^, which presented closer G ratios to those obtained in studies performed on irregular root canal anatomies ^9^ or artificial internal resorptive cavities.^24^ In fact, the current study exclusively quantified the ratio of filling materials and presence of V in the intrinsic anatomical irregularities and was not designed to evaluate root canal obturation in the main root canals. Accordingly, Marciano, et al.^25^ (2011) reported that the presence of isthmuses negatively affected the ratio of G in TH technique. This study also showed that among all irregularities, the worst obturation quality should be expected in accessory canals (M-LAC). Irrespective of the obturation technique used, a higher ratio of S than G should be foreseen for anatomical irregularities that are not treated with instruments.

 Differences in quality of obturation among techniques varied depending on the location of the anatomical irregularities. The core-carrier obturation techniques showed the highest ratio of G in the anatomical irregularities located at the apical third. In M-AI and M-IC, significantly higher G ratios were observed when GC or TH was used, followed by CW. This is in agreement with previous studies conducted on oval-shaped^26^ and C-shaped root canals.^9^ It is important to bear in mind that CW was performed at 175°C, instead of the recommended 200°C. Based on the manufacturer’s directions for use,^14^ it could be argued that this may reduce gutta-percha plasticization at the apical level. However, the temperature of 175°C is close to Buchanan^27^ (2004) recommendations, which stated that heating smaller pluggers (.04 and .06) to 185°C could be sufficient to achieve adequate filling at the apical third of the root canal.^28^ Moreover, it has been reported that classic ProTaper points show higher thermal conductivity, but less plasticity, and therefore less capacity to penetrate irregularities as compared to ProTaper G (Conform Fit™).^29^ Theoretically, this recent version use could have led to an even better performance of CW in apical third irregularities. Further research comparing traditional and more modern versions of gutta-percha is needed to validate this assumption.

CW achieved a significantly higher ratio of G than the other obturation techniques in the irregularities located in the coronal third, as previously reported in C-shaped canals,^9,30^ where the total area of the root canal is wider. This may be explained by the fact that in CW, there is an unlimited amount of G provided by injection system, in contrast to the limited amount provided by the obturator in core techniques.

 Voids were found in all the sections evaluated, with medians ranging from 0.01 to 0.15. The highest extent was detected in the apical irregularity (M-LACa) of LC groups and the lowest in coronal irregularities (D-IC) obturated with CW. Similar results were seen at the apical third of C-shaped canal replicas, with voids reaching up to 40% with CW and 22% with GC.^9^ Likewise, smaller percentages were found in the coronal third of mandibular molars when obturated with CW (2.86%).^31^ Similarly, 26% of V with TH has been reported in internal resorptions.^24^ At the same time, the presence of voids only differed significantly among obturation techniques in two irregularities (M-LI and M-IC) where the highest V values were detected in LC group. These results are in accordance with those reported by Marciano, et al.^25^ (2011) in the isthmuses of mesial root canals of mandibular molars.

## Conclusions

 Within the limitations of this *in vitro* study, it can be concluded that while CW is the most effective technique for the obturation of anatomical irregularities located in the coronal third of the root canal, core-carrier obturation techniques (TH and GC) achieve better quality of obturation in anatomical irregularities located at apical levels. LC obturation technique performs the worst in the presence of anatomical irregularities. Moreover, the worst quality of obturation should be expected in loop accessory canals, regardless of the obturation technique used.

## Data Availability

The datasets generated and analyzed during the current study are available from the corresponding author on reasonable request.
